# A pilot study to assess a novel screening questionnaire for central sleep apnea in patients undergoing overnight diagnostic polysomnography

**DOI:** 10.1007/s11325-026-03715-x

**Published:** 2026-05-23

**Authors:** Amanpreet Kaur, Asfandyar Ibrar, Ali Nadhim, Francisco Marquez, Kimberly Persaud, Bianca Dubovan, Ahmad Alkhatatneh, Mani Paliwal, Mary Grove, Jasmyne Rian Charles, David Goldstein, Florian P. Thomas, Divya Gupta

**Affiliations:** 1 Department of Neurology, Hackensack Meridian School of Medicine, Nutley, NJ USA; 2 JFK University Medical Center, JFK Neuroscience Sleep Center, Edison, NJ USA; 3 Hackensack Meridian Health, Edison, NJ USA; 4 Department of Neurology, Hackensack University Medical Center, Hackensack, NJ USA; 5 Hackensack Meridian School of Medicine, Department of Medical Sciences, Nutley, NJ USA

**Keywords:** Sleep apnea screening questionnaire, Heart failure, Cheyne-Stokes respiration, Central apnea index, Central sleep apnea, Atrial fibrillation

## Abstract

**Purpose:**

A novel central sleep apnea (CSA) screening questionnaire, SCOUTS-BAG-HARMS (SBH), was developed and assessed with polysomnography (PSG)-confirmed “any CSA”, defined as a central apnea index (CAI) ≥ 5 per hour. Unlike the STOP-BANG questionnaire, which screens for obstructive sleep apnea (OSA), SBH incorporates CSA-specific questions in three sections (SCOUTS), including silent apneas, demographics (BAG), including body mass index, and comorbidities (HARMS), including heart failure.

**Methods:**

In this pilot study, patients undergoing a PSG at our sleep lab between January and June 2023 were administered both the STOP-BANG and the SBH questionnaires. Questionnaire scores were compared to the CAI on in-lab PSG. Subset analysis assessed whether specific questions or sections of the SBH questionnaire have significant correlation with the finding of CAI ≥ 5 per hour.

**Results:**

Neither the STOP-BANG questionnaire nor the SBH questionnaire total score correlated linearly with the severity of CAI on PSG, but the SBH score ≥ 6 out of 20 correlated with the finding of CSA. On subset analysis, the HARMS section of SBH correlated with the finding of CSA on PSG. Adding the HARMS question responses to the total score on STOP-BANG also correlated with the finding of CSA.

**Conclusion:**

Since most patients with CSA also have some degree of obstructive type respiratory events, adding HARMS questions to the widely used STOP-BANG screening questionnaire for OSA may help identify patients at risk for CSA in the general population. This, in turn, would lead to prompt diagnostic testing and treatment for CSA.

## Introduction

Central sleep apnea (CSA) is characterized by repetitive cessation or decrease of both airflow and ventilatory effort during sleep due to intermittent cessation of the central respiratory drive. It is, therefore, often referred to as “silent apnea.” The resulting hypoxia stimulates chemoreceptors, leading to increased sympathetic activity [1] and a surge in blood pressure [2, 3], which increases cardiac muscle workload [4, 5] and reduces sleep quality.

The prevalence of central sleep apnea is high among patients with heart failure,

with estimates ranging from 18% to 25% [5–7]. Several studies have demonstrated a link between CSA and poor outcomes in patients with heart failure, including an effect on mortality [7, 8]. In a meta-analysis [9] CSA prevalence in stroke patients was around 7%, and it could be a consequence of involvement of the respiratory centers in the medulla. Sleep-related breathing disorders are an independent prognostic factor related to mortality after a first episode of stroke [10]. In a systematic review of CSA and chronic opioid use with eight studies comprising 560 patients, the mean prevalence of CSA was 24% [11]. This was a dose-dependent relationship; CSA was more prevalent in patients who chronically used opioids (70% vs. 5.0%) and more frequent (92%) at a morphine dose equivalent of 200 mg or higher [12]. In contrast, obstructive sleep apnea (OSA) is characterized by intermittent cessation of airflow during sleep due to the periodic collapse of the upper airway. OSA can cause snoring and daytime fatigue and may also lead to adverse cardiovascular outcomes.

Both OSA and CSA are diagnosed with a diagnostic polysomnography (PSG) test in the sleep lab. According to the AASM International Classification of Sleep Disorders (ICSD-3) [13], Central Sleep Apnea (CSA) is defined by ≥ 5 central apneas/hypopneas per hour of sleep, with the total number of central apneas and /or central hypopneas comprising > 50% the total apnea-hypopnea index (AHI). However, a diagnosis of CSA with Cheyne Stokes Breathing (CSB) does not exclude a diagnosis of OSA. In fact, in patients with OSA and CSA-CSB, the relative amount of central and obstructive apnea can vary over time or even within the same night.

**Fig. 1:**
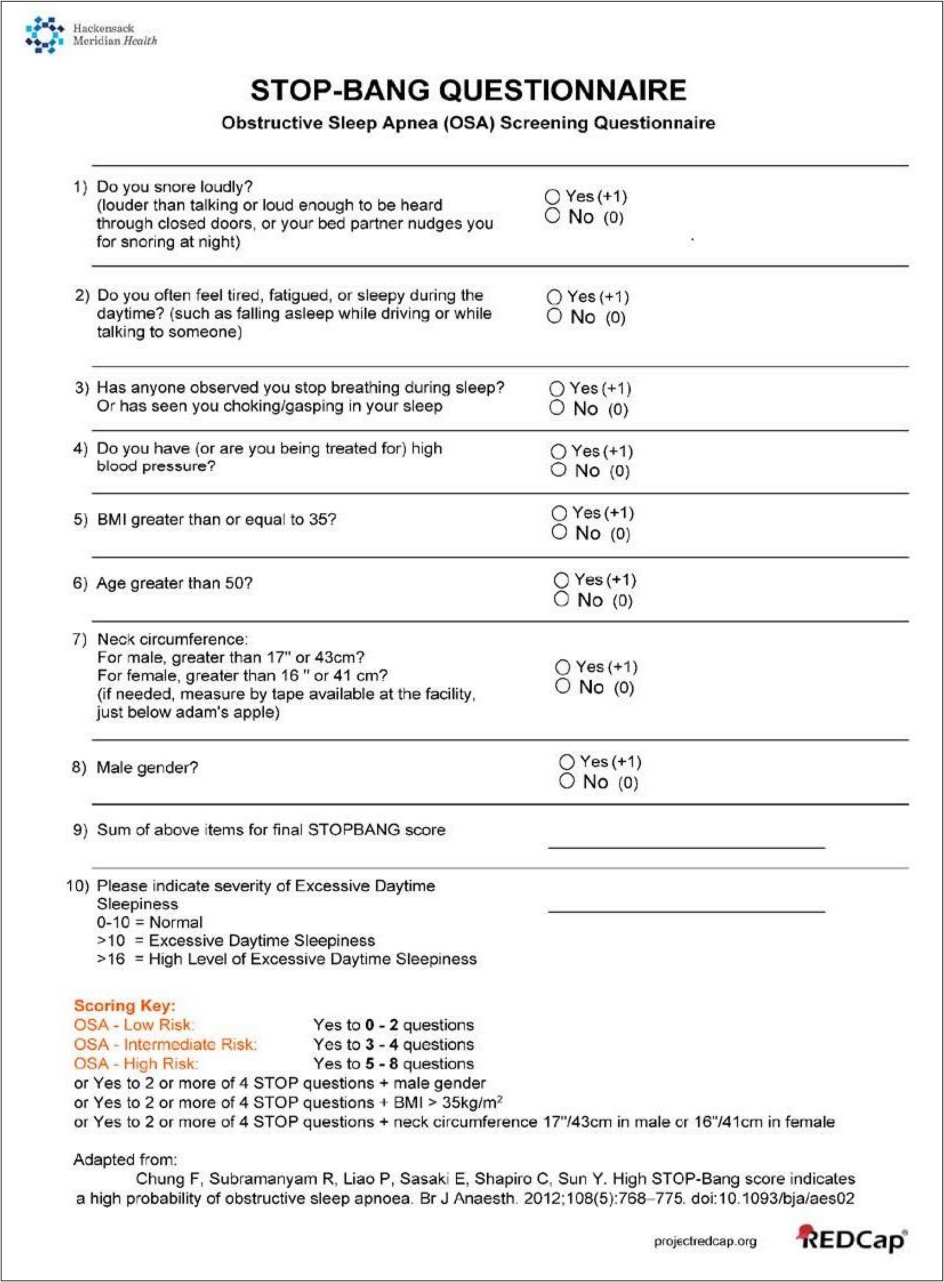
STOP-BANG questionnaire. Screening tool for obstructive sleep apnea (OSA), adapted from Chung et al. A high STOP-BANG score indicates a high probability of OSA. STOP-BANG = snoring, tiredness, observed apnea, high blood pressure, body mass index, age, neck circumference, and gender

The STOP-BANG questionnaire, shown in Fig. 1, is a widely used screening tool for OSA. It includes: (S)noring, feeling (T)ired, a history of (O)bserved apneas, a history of high blood (P)ressure, associated with class II obesity or (B)MI > 35, with (A)ge > 50 years, with (N)eck circumference > 40 cm, and with male (G)ender. However, to date, a cost-effective and convenient screening tool for the early detection of CSA is not available. While symptoms of CSA like nocturnal awakenings, witnessed apneas, nocturia are similar to OSA, the salient differences are that CSA patients may not report snoring or daytime sleepiness, and rather they may have daytime fatigue and difficulty concentrating [14, 15]. Of note, the sleep heart health study data analysis to compare the clinical features of CSA versus OSA, revealed that the subjects with CSA had higher age, lower BMI, lower scores on Epworth Sleepiness Scale and were more likely to be male [16]. Also, the prevalence of CSA and Cheyne-Stokes respiration (CSR) in this sample was low, at 0.9 and 0.4. Even amongst subjects with heart failure, prevalence of OSA was higher at 55%, than of CSA at 4.1%. Hence, given its variation in symptoms from the standard screening questionnaire and its low prevalence, CSA may be missed in routine clinical encounters. Albeit CSA testing and treatment would help to improve cardiovascular outcomes and quality of life for patients.

The purpose of this pilot study is to investigate the relationship between the score of our proposed SBH screening questionnaire to the findings of “any CSA” as determined by PSG. Specifically, we hypothesize that a score of 6 or more out of 20 on the SBH questionnaire will be associated with a CAI ≥ 5 per hour on the diagnostic PSG.

**Primary Aim**:

- To determine the relationship between the SBH questionnaire score ≥ 6and
- a PSG CAI ≥ 5 per hour, indicating the presence of any CSA

**Secondary Aims**:

1. To evaluate the SBH questionnaire for risk stratification, with the hypothesis that a higher total score predicts a higher risk of CAI *≥* 5 per hour.
2. To conduct a subset analysis of the SBH questionnaire items to identify those questions or sections that are more predictive of any CSA.

## Methods

**Fig. 2:**
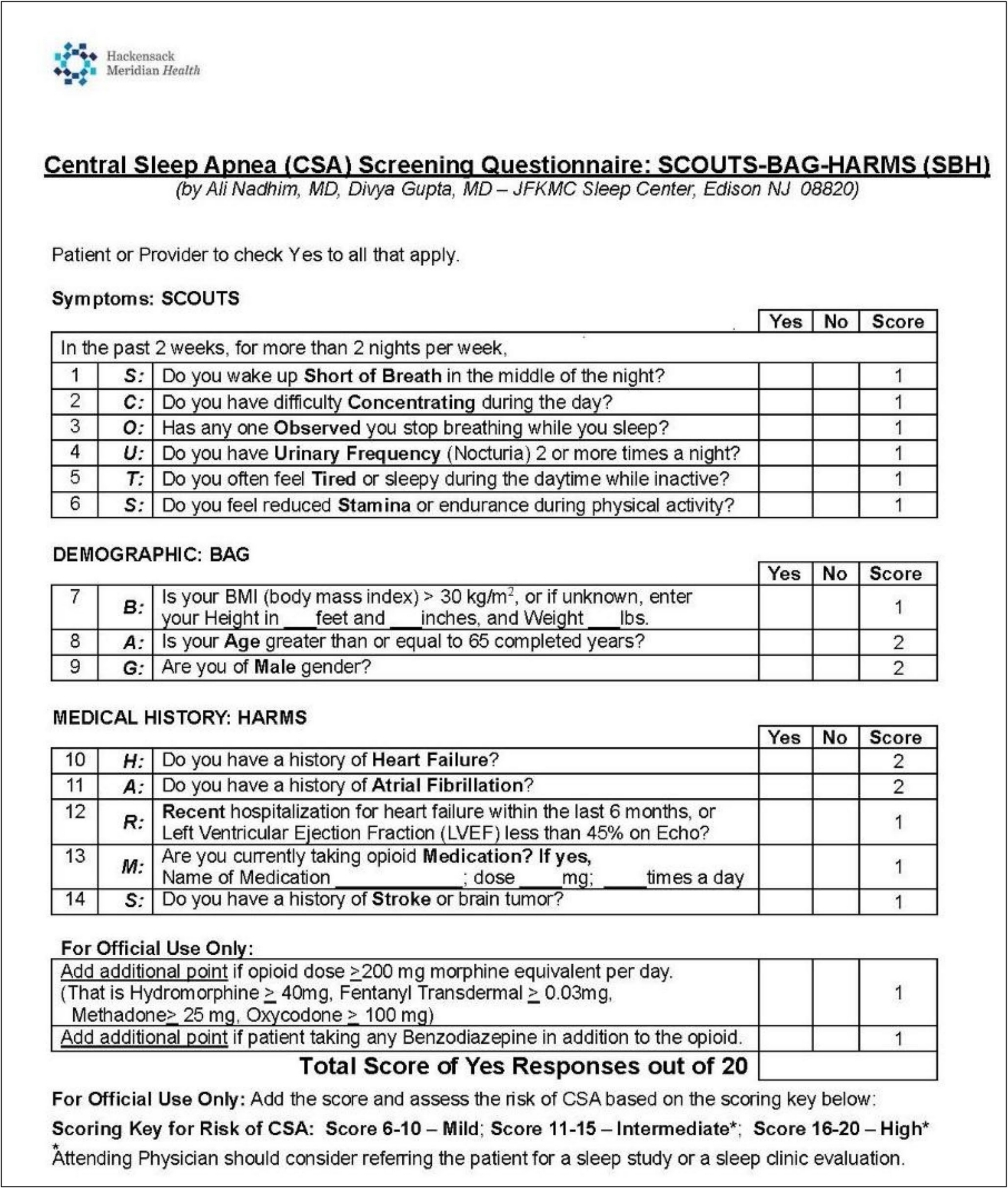
SCOUTS-BAG-HARMS questionnaire developed for screening central sleep apnea

The SBH research questionnaire was developed by our study team at our academic sleep center in central New Jersey, based on the latest evidence. As shown in Fig. 2, the symptoms, demographics, and comorbidities commonly associated with CSA were clustered under the sections of SCOUTS, BAG, and HARMS, respectively, to develop this acronym. This structure also helps identify which questions are more strongly linked to any CSA, which in turn could support the development of a weighted scoring system.

SCOUTS represents symptoms including shortness of breath during sleep, difficulty concentrating, observed apneas, nocturia (> 2 times/night), daytime fatigue, and reduced stamina.

BAG represents demographic variables including body mass index (BMI > 30), age > 65 years, and male gender. HARMS represents comorbid conditions including history of heart failure, atrial fibrillation, recent hospitalization for heart failure within six months or LVEF < 45%, opioid medication use, and history of stroke.

The SCOUTS-BAG-HARMS included: (S)hortness of breath in the middle of the night, difficulty (C)oncentrating during the day, (O)bserved apneas during sleep, (U)rinary frequency > 2 times per night, feeling (T)ired during the day, a feeling of reduced (S)tamina during physical activity, (B)MI > 30, (A)ge > 65 years, male (G)ender, a history of (H)eart failure, a history of (A)trial fibrillation, (R)ecent hospitalization for heart failure within the last 6 months or LVEF < 45% on most recent echocardiogram, current opiate (M)edication and a history of stroke (S). Compared to the STOP-BANG screening questionnaire for OSA, the SCOUTS-BAG- SBH excludes snoring and includes silent apneas, a lower Body Mass Index (BMI), and medical comorbidities associated with CSA.

Initial psychometric validation was conducted in the sleep lab by piloting the questionnaire with 10 subjects similar to the study population and collecting a post-questionnaire feedback survey. The revised version was subsequently used for the data validation part of the study. Notably, most patients with CSA exhibit some degree of obstructive type respiratory events as well. Therefore, in our study, both screening questionnaires, SBH and STOP-BANG, were administered to the study subjects, and response scores for each questionnaire were correlated with the PSG outcomes, including the CAI, for data validation.

### Patient selection

This prospective observational pilot study included all patients aged 21 years or older who presented for a diagnostic PSG test at our sleep lab. Exclusion criteria included airway cancer or recent upper airway surgery. Patients were withdrawn from the study if they were unable to complete the PSG test after filling out the questionnaire, and no further data were collected on them.

### Questionnaire administration

All of the study tools were built in REDCap [17], a secure web-based application for research, supported by our healthcare network. The night sleep technologist first obtained signed informed consent from the patient, then, within our REDCap study project, created a record identification number and administered both questionnaires.

### Diagnostic sleep test (PSG) recording and scoring

Thereafter, the patient’s PSG was recorded on the software for PSG acquisition. Later, each PSG recording was scored by a daytime sleep technologist using the AASM adult scoring criteria to assess the patient’s total apnea-hypopnea index (AHI) and it’s component of CAI. Accordingly, respiratory events lasting ≥ 10 s with ≥ 90% drop in airflow were scored as apneas, while those with ≥ 30% reduction in airflow were scored as hypopneas if accompanied by ≥ 3% oxygen desaturation and/or an arousal (“recommended” criteria for hypopneas). Apneas were further classified as Obstructive (if there was continued respiratory effort), or Central (if there was lack of respiratory effort during the event). For this study, we did not further classify hypopneas as obstructive versus central due to poor interscorer reliability of central hypopneas. Therefore, central apnea index (CAI), rather than the central apnea-hyoponea index (CAHI) of the patient’s PSG, was selected as the outcome to be correlated with their score on the SBH questionnaire. So, the SBH score was correlated with patients with CAI ≥ 5 per hour (Any CSA), rather than the standard definition of CSA per se, as defined in the ICSD-3. The PSG was then reviewed and finalized by sleep medicine physicians, and the report was uploaded into the patient’s electronic health record (EHR). The scoring technologist and the interpreting physician of the PSG were blinded to the study participants and their questionnaire responses.

### PSG data collection

To complete the data set for each patient in REDCap, the study staff reviewed the finalized PSG report from the patient’s EHR and populated the PSG outcomes into the REDCap record for that patient.

### Statistical analysis

All STOP-BANG and SBH total scores were included in our study and analyzed in conjunction with the corresponding CAI on the patient’s overnight diagnostic PSG. Data was analyzed using SPSS 22.0 (IBM Corp). All demographic data were analyzed using descriptive statistics and presented as the mean (M) ± standard deviation (SD) for continuous variables and as N (%) for categorical variables. Parametric and non-parametric *t*-test, Pearson Correlation, Chi-square test and Binomial logistic regression were used to assess relationships between the scores. All *p* < 0.05 were considered significant.

## Results

Between January and June 2023, 415 consecutive patients presented for diagnostic PSG to the sleep Lab, out of which 280 consented to participate in the study, and 262 completed the questionnaires. 53% were males, 35% were more than 65 years of age, and 64% were above the age of 50 yrs. 45% of the patients’ BMIs were higher than 30. The mean (SD) score for the SBH was 5.91 (2.47), the mean (SD) score for the STOP-BANG + HARMS was 4.7 (2.1), and the mean (SD) score for the STOP-BANG was 4.03 (1.58).

Out of these 262 patients, 27 had the finding of “any CSA”, which for the purposes of this study of this study was defined as CAI ≥ 5 per hour of total sleep time. Out of these 27 patients, 7 had the finding of “predominant CSA”, rather than OSA, defined as a CAI ≥ 5 per hour plus CAI representing ≥ 50% of the total apnea-hypopnea index (AHI) on their diagnostic PSG. For any CSA patients (*n* = 27), the subset analysis revealed that 74% were male, 55% were 65 years of age or older, and 69% were above the age of 50. 37% of the patients’ BMIs were higher than 30. The mean (SD) score for the SBH was 7.29 (2.39), the mean (SD) score for the STOP-BANG + HARMS was 5.68 (2.61), and the mean (SD) score for the STOP-BANG was 4.24 (1.94). For predominant CSA patients (*n* = 7) compared to the other any CSA patients, the subset analysis showed fewer male patients (43%), older age (57% were above 65 years of age and 83% were above 50 years of age) and less obesity (BMI > 30 in 28.6% of patients).

### Correlation analysis

Correlation analysis was performed to see if there is any relationship between the CAI and Total AHI on PSG, with the response scores on SBH questionnaire, STOP-BANG questionnaire, SCOUTS section, BAG section, HARMS section, and the STOP-BANG + HARMS hybrid score, as shown in Table 1. There was a statistically significant positive relationship between CAI & SBH (*p* < 0.005), CAI & BAG (*p* < 0.005), CAI & HARMS (*p* < 0.005), and CAI and STOP-BANG + HARMS (*p* < 0.005).

Additionally, there was a statistically significant positive relationship between total AHI and SBH (*p* < 0.005), total AHI and STOP-BANG (*p* < 0.005), total AHI and SCOUTS (*p* < 0.05), total AHI and BAG (*p* < 0.005), total AHI and HARMS (*p* < 0.05), and total AHI and STOP-BANG + HARMS (*p* < 0.005), as shown in Table 1.

**Table 1:** Pearson correlation coefficients and *p*-value for associations between sleep-related indices (CAI, AHI) and risk assessment tools (SBH, STOP-BANG, SCOUTS, BAG, HARMS, and Combined STOP-BANG + HARMS)

	SBH	STOP-BANG	SCOUTS	BAG	HARMS	STOP-BANG + HARMS
CAI						
Pearson Correlation	0.287^**^	0.064	-0.016	0.217^**^	0.347^**^	0.270^**^
*p*-value (2-tailed)	0.000	0.320	0.805	0.001	0.000	0.000
Total AHI						
Pearson Correlation	0.334^**^	0.435^**^	0.147^*^	0.272^**^	0.151^*^	0.415^**^
*p*-value (2-tailed)	0.000	0.000	0.018	0.000	0.019	0.000

Significance levels: * *p* < 0.05. ** *p* < 0.01 (2-tailed)

Abbreviations: *CAI *central apnea index, *AHI* apnea-hypopnea index, *SBH* SCOUTS-BAG-HARMS, *STOP-BANG* Snoring, Tiredness, Observed apneas, high blood Pressure, BMI >35, Age >50y, Neck circumference, and Gender, *SCOUTS *Symptoms of CSA (snoring, choking, observed apneas, unrefreshing sleep, tiredness, shortness of breath), *BAG *BMI, Age, and Gender, *HARMS *composite of the following comorbidities: H, history of heart failure; A, atrial fibrillation; R, recent hospitalization for heart failure within 6 months; M, currently taking opioid medication; and S, history of stroke or brain tumor

### Chi-square tests for statistical significance

Among patients with any CSA (CAI ≥ 5 per hour), chi-square tests were conducted to examine associations between categorical variables. Statistically significant associations were observed between any CSA status and the following: STOP-BANG + HARMS score; STOP-BANG (≥ 4) + HARMS (≥ 1); STOP-BANG (≥ 3) + HARMS (≥ 1); BAG; HARMS; H (history of heart failure); R (recent hospitalization for heart failure within 6 months); and M (current opioid use) with *p* < 0.05 and *p* < 0.005. Phi and Cramer’s V also show weak to moderate strength of association, as shown in Table 2.

The proportion of patients with any CSA was 14.6% among those with a STOP-BANG + HARMS score of 4, 17.6% with a STOP-BANG ≥ 4 and HARMS ≥ 1, and 18.2% with a STOP-BANG ≥ 3 and HARMS ≥ 1. Among individual sections of the SBH questionnaire, any CSA was present in 37% of patients with a BAG score of 3, 16.7% with a HARMS score of 1, and 14.6% with a HARMS score of 2. For individual HARMS components, any CSA was identified in 25.0% of patients with a history of heart failure, 31.6% of those recently hospitalized for heart failure, and 30.0% of those currently taking opioid medication.

**Table 2:** Chi-Square *p*-value for associations between any CSA (CAI ≥ 5 per hour) and SCOUTS-BAG-HARMS components, STOP-BANG scores, and individual HARMS items and Phi/Cramer’s V for effect size

	Any CSA(CAI ≥5/h) Pearson Chi-Square	*p*-value (2-tailed)	Phi /Cramer’s V
SCOUTS-BAG-HARMS	17.124	0.194	0.277
SCOUTS	11.039	0.087	0.208
BAG	11.884	0.036*	0.225
HARMS	15.946	0.014*	0.256
STOP-BANG	11.113	0.195	0.213
STOP-BANG + HARMS	24.006	0.013*	0.320
STOP-BANG ( > = 4) + HARMS ( > = 1)	4.678	0.031*	0.140
STOP-BANG ( > = 3) + HARMS ( > = 1)	6.688	0.010*	0.167
HARMS: H (History of Heart Failure)	7.820	0.005*	0.176
HARMS: A (History of A-fib)	1.129	0.290	0.067
HARMS: R (Recent Hospitalization for HF within 6 months)	10.583	0.001**	0.204
HARMS: M (Currently taking Opioid Medication)	4.143	0.042*	0.127
HARMS: S (History of Stroke and/or Brain Tumor)	0.001	0.982	0.001
Combined H (History of HF) + R (Recent Hospitalization within 6 months)	2.763	0.096	0.104

Significance levels: * *p* < 0.05. ** *p* < 0.01 (2-tailed)

Abbreviations: *CSA* central sleep apnea, *CAI* central apnea index, *STOP-BANG* Snoring, Tiredness, Observed apneas, high blood Pressure, BMI > 35, Age > 50y, Neck circumference, and Gender, *SCOUTS* Symptoms of CSA (snoring, choking, observed apneas, unrefreshing sleep, tiredness, shortness of breath), *BAG* BMI, Age, and Gender, *HARMS* composite of the following comorbidities: H, history of heart failure; A, atrial fibrillation; R, recent hospitalization for heart failure within 6 months; M, currently taking opioid medication; and S, history of stroke or brain tumor

### *t*-test analysis

The Shapiro-Wilk test was conducted for all the scores, SBH, STOP-BANG, STOP-BANG + HARMS and Total HARMS. The result indicated a violation of normality for all scores, *p* < 0.05. The Levene’s test for homogeneity of variance indicated equal variances for SBH and STOP-BANG score, *p* > 0.05 and indicated a violation of equality of variances for STOP-BANG + HARMS and Total HARMS score, *p* < 0.05. The two groups, Any CSA (Yes/No), also had unbalanced sample sizes.

Given the unequal variances and unbalanced sample sizes, we selected Welch’s *t*-test to compare the group means as our primary statistical approach. This test was chosen because it directly addresses our aim concerning the difference in means and is robust to the observed violation of homogeneity of variance. However, given that the data also deviated from a normal distribution, we conducted a secondary, non-parametric sensitivity analysis using the Mann-Whitney *U* test. This was done to ensure that our findings were robust and not dependent on the normality assumption of the primary test.

As shown in Table 3, the Welch’s *t*-test indicated a significant difference in the means of SBH, and HARMS scores between the two groups (*p* < 0.05). No significant difference was found in the mean STOP-BANG score, and STOP-BANG + HARMS scores (*p* > 0.05).

These findings were supported by a non-parametric Mann-Whitney *U* test, which also revealed a significant difference between the groups for SBH score (*p* < 0.05). No significant difference was found in the STOP-BANG score, and STOP-BANG + HARMS scores (*p* > 0.05). The exact *p*-value for the Mann-Whitney *U* test could not be computed for the HARMS score, likely due to the small sample size.

**Table 3:** Difference in group means and *p*-value for Welch’s *t*-test and for Mann-Whitney *U* test. Any CSA (CAI ≥ 5 per hour) group (Yes/No) for SBH, STOP-BANG + HARMS, HARMS, STOP-BANG scores

	Any CSA Group (Yes) - Mean	Any CSA Group (No) - Mean	*p*-value Welch’s t-test(2-tailed)	*p*-value Mann-Whitney*U* test (2-tailed)
SBH	7.292	5.729	0.005	0.004
HARMS	1.417	0.575	0.023	-
STOP-BANG	4.240	4.018	0.586	0.760
STOP-BANG + HARMS	5.682	4.599	0.071	0.070

The mean SBH score was 5.7 for patients without Any CSA and 7.3 for those with Any CSA

Abbreviations: *CSA* central sleep apnea, *SBH* SCOUTS-BAG-HARMS, *STOP-BANG* Snoring, Tiredness, Observed apneas, high blood Pressure, *BMI > 35* Age > 50, Neck circumference, and Gender, *HARMS* composite of the following comorbidities: H, history of heart failure; A, atrial fibrillation; R, recent hospitalization for heart failure within 6 months; M, currently taking opioid medication; and S, history of stroke or brain tumor

### Binomial logistic regression analysis

Binomial logistic regression was used to determine if any of the scales/scores or categorical variables (Yes/No) predicted the outcome variable: any CSA. Several clinical variables and composite scores were assessed as predictors of any CSA using binomial logistic regression. The SCOUTS-BAG-HARMS (SBH) score, STOP-BANG + HARMS score, and total HARMS score were all significantly associated with increased likelihood of any CSA (*p* < 0.005, *p* < 0.05, and *p* = 0.005). These scores explained 7.5%, 4.6%, and 6.6% of the variance in any CSA, respectively.

Dichotomized versions of the STOP-BANG + HARMS score of ≥ 4 and ≥ 3 on STOP-BANG combined with ≥ 1 on HARMS were also significant predictors (*p* < 0.05 and *p* < 0.05), with odds ratios of 2.63, 95% CI (1.07, 6.49), *p* < 0.05 and odds ratio of 2.98, 95% CI (1.26, 7.03), *p* < 0.05, respectively. These two combinations explained 3.7% and 5.2% of the variance in any CSA, respectively. Within the HARMS score components, history of heart failure (HF) and recent HF hospitalization (within 6 months) were individually associated with a significantly increased likelihood of any CSA (*p* < 0.05 and *p* = 0.006), with odds ratios of 3.53, 95% CI (1.39, 8.92), *p* < 0.005 and odds ratio of 5.14, 95% CI (1.75, 15.11), *p* < 0.005, respectively. These two individual components explained 5.0% and 5.9% of the variance in any CSA. Also, the entire 95% CI is above 1.0 for all the predictors above, indicating statistically significant odds of predicting any CSA.

While a chi-square test suggested a possible association between opioid medication use and any CSA, this was not confirmed by logistic regression. Neither history of atrial fibrillation nor history of stroke/brain tumor showed a significant association with any CSA in chi-square analyses, precluding further logistic regression assessment.

The ability of various scores to discriminate between individuals with and without any CSA was assessed using area under the receiver operating characteristic curve (AUC) analysis. The STOP-BANG score demonstrated poor discriminatory ability, with an AUC of 0.341, suggesting performance only slightly better than chance. Exploring different cut-off points for the STOP-BANG score (2.5 and 3.5) revealed a trade-off between sensitivity (0.6 − 0.4) and specificity (1-specificity: 0.842 − 0.598), but overall performance remained suboptimal.

**Fig. 3:**
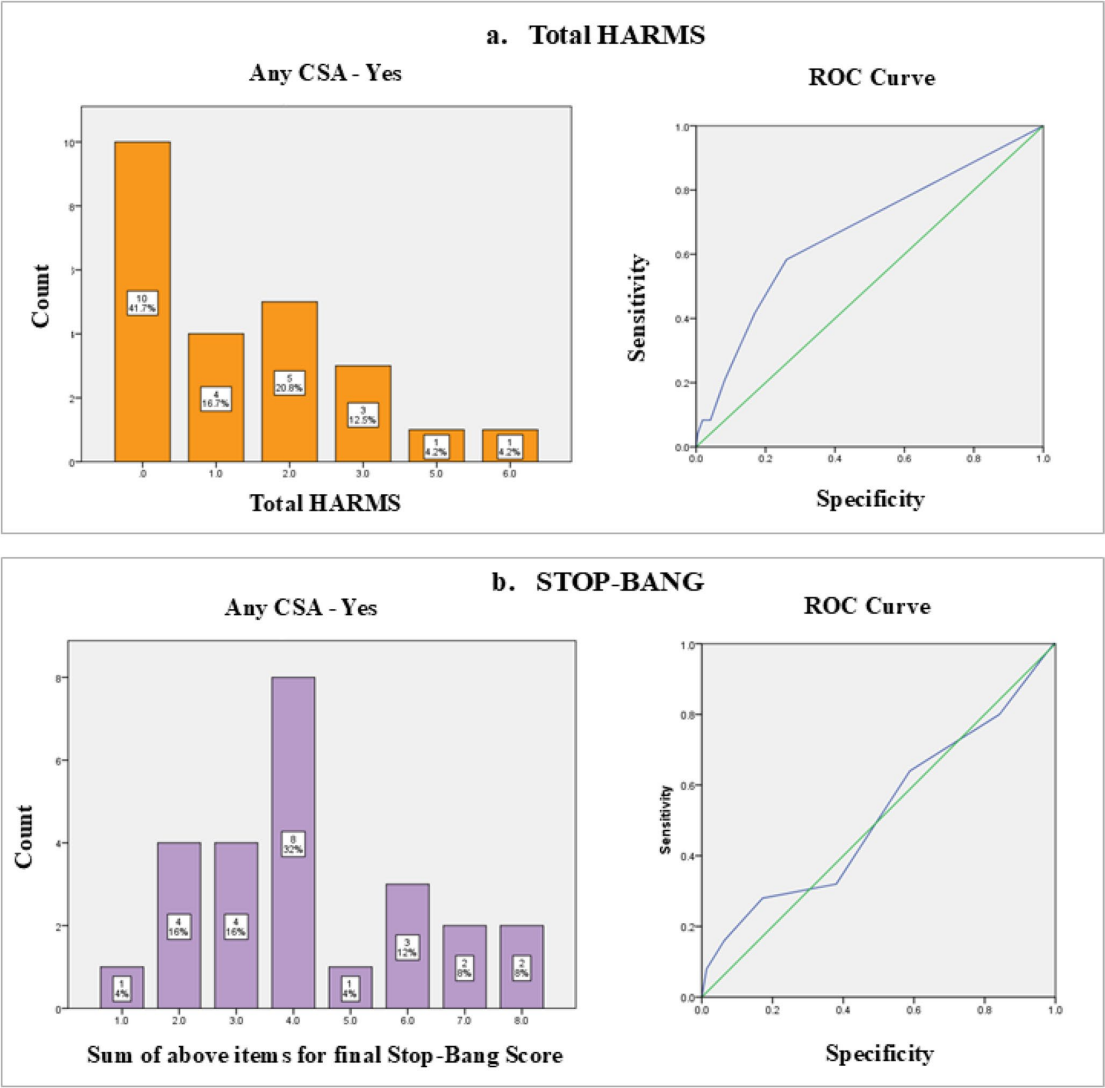
Frequency distribution and receiver operating characteristic (ROC) curves for STOP-BANG score (**a**) and total HARMS score (**b**). Deflection points indicate sensitivity and 1-specificity cut-off values for CSA on polysomnography (PSG). Any CSA (CAI *≥* 5 per hour), HARMS = heart rate variability, atrial fibrillation, reduced ejection fraction, male sex, supine sleep position; CSA = central sleep apnea; ROC = receiver operating characteristic; PSG = polysomnography

The total HARMS score demonstrated the best discriminatory ability among the evaluated scores, with an AUC of 0.670, which is modest and likely due to the small sample size of our study. Cut-off points of 0.5 and 1.5 for the total HARMS score yielded sensitivities ranging from 0.571 to 0.429 and specificities (1 - specificity) ranging from 0.284 to 0.186, as presented in Fig. 3.

**Fig. 4:**
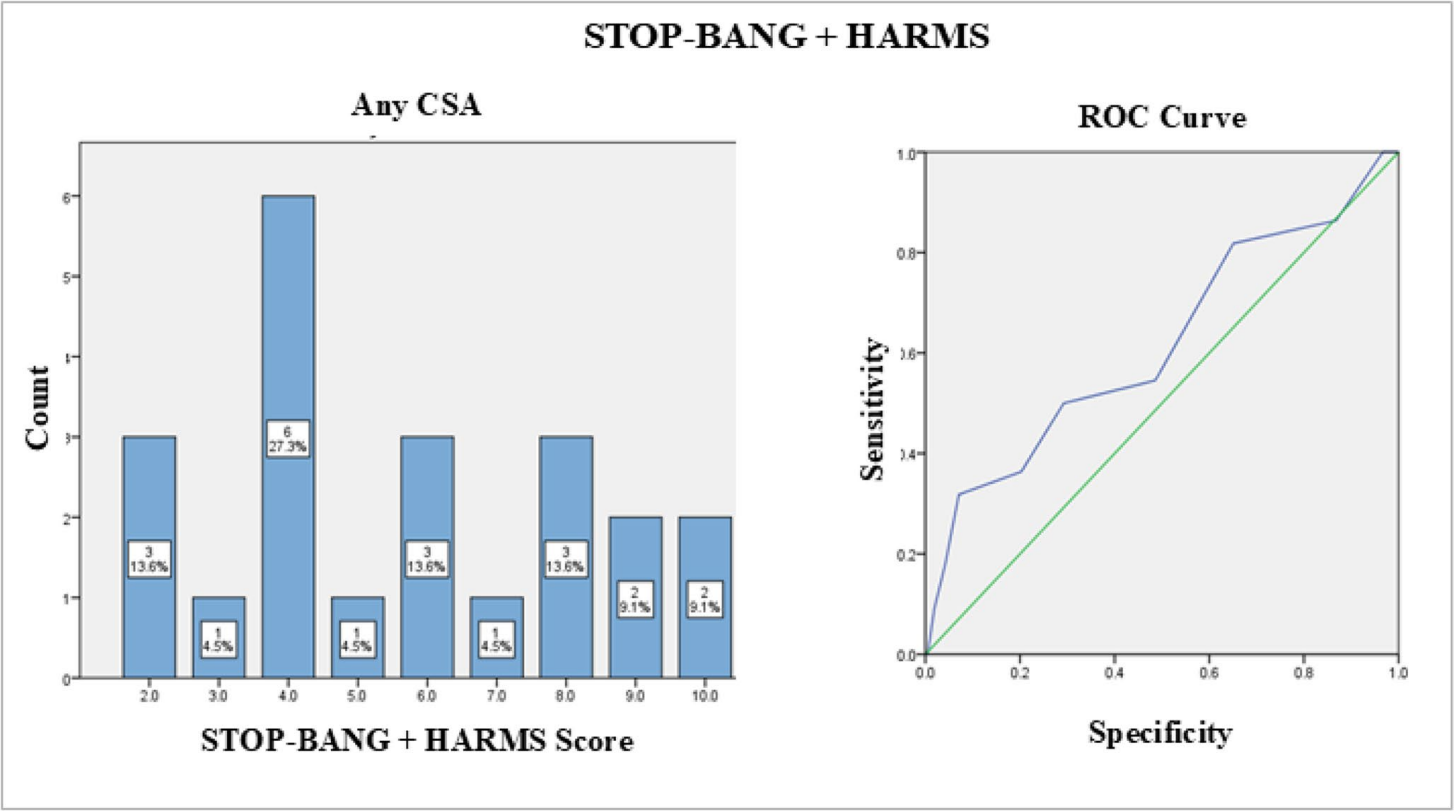
Frequency distribution and ROC curve for combined STOP-BANG + HARMS score. Deflection points indicate sensitivity and 1-specificity cut-off values for CSA on PSG. Any CSA (CAI *≥* 5 per hour); ROC = receiver operating characteristic; CSA = central sleep apnea; PSG = polysomnography

The combined STOP-BANG (8) + HARMS (7) score showed slightly improved discriminatory ability, with an AUC of 0.568. At a cut-off point of 3.5, the combined score achieved a sensitivity of 0.8 but at the cost of lower specificity (1 - specificity: 0.664), as depicted in Fig. 4.

### Descriptive statistics

Table 4 shows the total number of patients with H, A, R, M, S, and the total number of patients with any CSA, and the mean scores for STOP-BANG and STOP-BANG + HARMS for these patients. In the subset analysis, as shown in Table 2, the association of the SCOUTS (symptoms) section of the SBH with any CSA was not statistically significant (*p* > 0.05), but the BAG (demographics) section of the SBH was significantly associated with any CSA (*p* < 0.05) and the HARMS (comorbidities) section of the SBH was significantly associated with any CSA (*p <* 0.05).

**Table 4:** Descriptive statistics for individual HARMS components in relation to any CSA (CAI ≥ 5 per hour) status and mean STOP-BANG and STOP-BANG + HARMS scores

	Number (n) out of 262 total pts	Any CSA pts=27	STOP-BANG score(mean) of n pts	STOP-BANGscore (mean) Any CSA pts	STOP-BANG + HARMS score (mean) n pts	STOP-BANG+HARMS score(mean)Any CSA pts
H: Heart Failure	32	8/32	4.58	4.71	7.9	8.0
A: A-Fib	27	4/27	3.89	3.75	7.1	7.75
R: Recent Hospitalization	19	6/19	3.63	2.83	6.82	6.0
M: Opioid Med (without MME)	10	3/10	3.9	4.67	6.75	8.5
S: Stroke	29	3/29	4.21	4.33	6.2	7.0
CSA pts = 27” refers to patients with any CSA. “n pts” refers to the number of patients with the specified HARMS condition. All scores reflect mean values within the specified subgroups						

*Abbreviations: CSA* central sleep apnea, *CAI* central apnea index, *STOP-BANG* Snoring, Tiredness, Observed apneas, high blood Pressure, BMI > 35, Age > 50 y, Neck circumference, and Gender, *HARMS *composite of the following comorbidities: H, history of heart failure; A, atrial fibrillation; R, recent hospitalization for heart failure within 6 months; M, currently taking opioid medication; S, history of stroke or brain tumor

## Discussion

Improving risk assessment tools and screening protocols may help with the early detection of CSA, leading to prompt testing and treatment and better health outcomes. This study examined several significant predictors of central sleep apnea (CSA), including composite scores such as the SCOUTS-BAG-HARMS and STOP-BANG + HARMS, as well as individual risk factors like a history of HF and recent HF hospitalization. The strong association between HF and any CSA observed in this study aligns with existing literature [18, 19]. Patients with predominantly CSA on polysomnography have been shown to be older and to have more advanced heart failure, as reflected by higher New York Heart Association class, compared with those with OSA [18]. Additionally, our findings are consistent with a large cohort study of patients undergoing polysomnography, which demonstrated that individuals with CSA had a much greater burden of cardiovascular comorbidities, including myocardial infarction, atrial fibrillation, stroke, and heart failure. Patients with OSA were intermediate, while those without sleep-disordered breathing (SDB) had the fewest cardiovascular comorbidities (*p* < 0.001 for all comparisons) [19]. Hence, the particularly high odds ratio associated with recent HF hospitalization in our study further emphasizes the need for increased vigilance in screening for CSA after such episodes.

The mean SBH score was higher in patients with any CSA (7.3) compared to those without any CSA (5.7). Given the small sample size, these are preliminary findings and support our initial hypothesis that an SBH score ≥ 6 out of 20 may be a reasonable threshold for identifying patients at risk for any CSA. In subset analysis, the BAG (demographics) and HARMS (comorbidities) sections of the SBH questionnaire were significantly associated with any CSA, whereas the SCOUTS (symptoms) section was not, as shown in Table 1. These findings suggest that comorbid conditions may be more predictive of any CSA than symptom-related responses and should be considered for diagnostic sleep testing rather than relying solely on symptoms of SDB or demographic characteristics, such as BMI and gender. Similar findings have been reported for the widely used STOP-BANG screening questionnaire for OSA, in that the BANG questions (pertaining to demographics) demonstrated greater predictive value than the STOP questions (pertaining to symptoms), and the use of a weighted score with higher emphasis on the BANG questions has been shown to maintain sensitivity of the questionnaire while improving specificity [20]. The lower predictive value of symptoms on these questionnaires could be possibly due to underestimation of symptoms by patients during their sleep, and its reliance on observer reports that are not consistently available.

As most patients with CSA also have obstructive type respiratory events in varying degrees, both SBH and STOP-BANG screening questionnaires were administered to the study subjects, and response scores of each questionnaire were correlated with the PSG outcomes for CAI. Our findings also indicate that the total score on either of these questionnaires, STOP-BANG or SBH, was not linearly associated with the severity of the central apnea index on the PSG.

Of note, a STOP-BANG score of 3 or 4 indicates intermediate risk for OSA, and supportive information is required to determine whether to refer a patient for a diagnostic test. Since the ROC curve showed moderate sensitivity and specificity near the score of 4, we chose a STOP-BANG cut-off score of 4 for the analysis. In the case of Total HARMS, we chose a cut-off score of 1 for the analysis as the ROC curve showed moderate sensitivity and specificity near the score 1. Since a score of ≥ 4 on STOP-BANG, plus a score of ≥ 1 on Total HARMS, was significantly associated with the finding of any CSA, as shown in Table 2, we suggest including the HARMS section along with STOP-BANG when screening patients to determine the need for further diagnostic testing. The presence of this association may lead to diagnostic PSG testing in patients with an intermediate STOP-BANG score who might otherwise be missed for further evaluation. Therefore, the use of this expanded STOP-BANG plus HARMS questionnaire may enhance its ability to assess the risk of any CSA.

### Limitations

The pilot study was conducted at a single center, and the sample size was limited. Also, the psychometric validation could have been more robust. Therefore, the findings should be interpreted as preliminary. The relatively small number of patients with predominant CSA made the risk stratification of the SBH questionnaire insufficient for analysis purposes, but it is in alignment with the relatively lower prevalence of predominant CSA in patients at risk of SDB [16]. Additionally, although a Spanish translation was available, most of the questionnaires were completed in English, which limits the generalizability to non-English speaking populations. Future studies could also conduct more extensive psychometric validation.

Regarding each distinct item within the HARMS section of the questionnaire, the apparent lack of association with history of atrial fibrillation, stroke, and/or brain tumor may be artefactual due to their limited sample size in our study population. Also, the discrepancy between the chi-square test and logistic regression results for opiate use may be due to similar reasons. However, this also presents an opportunity for future research, specifically focused on HARMS questions to further characterize the association of any CSA with these conditions.

## Conclusions

Preliminary findings from our study indicate that since most patients with CSA also have some degree of obstructive type respiratory events, adding HARMS questions to the widely used STOP-BANG screening questionnaire for OSA may help identify patients at risk for CSA in the general population. This could facilitate earlier diagnostic testing and treatment for CSA. Developing a rapid, reliable, and accurate CSA screening tool for clinicians could support the early detection of CSA in high-risk patient populations, ultimately improving patients’ sleep and quality of life while reducing the risk of adverse health outcomes. Over time, this approach could lead to more efficient use of healthcare resources. Further studies with larger sample sizes are needed to validate the SBH.

## Data Availability

The datasets generated and/or analyzed during the current study are available from the corresponding author upon reasonable request.
